# Deep Learning Regression Approaches Applied to Estimate Tillering in Tropical Forages Using Mobile Phone Images

**DOI:** 10.3390/s22114116

**Published:** 2022-05-28

**Authors:** Luiz Santos, José Marcato Junior, Pedro Zamboni, Mateus Santos, Liana Jank, Edilene Campos, Edson Takashi Matsubara

**Affiliations:** 1 Faculty of Computer Science, Federal University of Mato Grosso do Sul, Campo Grande 79070-900, MS, Brazil; luiz.h.s.santos@ufms.br (L.S.); edilene.veneruchi@ufms.br (E.C.); 2 Faculty of Engineering, Architecture and Urbanism and Geography, Federal University of Mato Grosso do Sul, Campo Grande 79070-900, MS, Brazil; jose.marcato@ufms.br (J.M.J.); pedro.zamboni@ufms.br (P.Z.); 3 Embrapa Beef Cattle, Brazilian Agricultural Research Corporation, Campo Grande 79106-550, MS, Brazil; mateus.santos@embrapa.br (M.S.); liana.jank@embrapa.br (L.J.)

**Keywords:** regrowth density, deep learning, forages

## Abstract

We assessed the performance of Convolutional Neural Network (CNN)-based approaches using mobile phone images to estimate regrowth density in tropical forages. We generated a dataset composed of 1124 labeled images with 2 mobile phones 7 days after the harvest of the forage plants. Six architectures were evaluated, including AlexNet, ResNet (18, 34, and 50 layers), ResNeXt101, and DarkNet. The best regression model showed a mean absolute error of 7.70 and a correlation of 0.89. Our findings suggest that our proposal using deep learning on mobile phone images can successfully be used to estimate regrowth density in forages.

## 1. Introduction

Pasture areas cover 21% of the territory (170 million hectares) in Brazil; however, a large part of these pastures are degraded [1], leading to lower livestock productivity. The current average Brazilian productivity (73.5 kg of CWE. ha${}^{-1}$·yr${}^{-1}$) is lower than the potential productivity of 294 kg CWE.ha${}^{-1}$·yr${}^{-1}$ [2]. This production gap represents a great challenge to be surpassed by the livestock producing countries. On one hand, the increase in the world population leads to increased demand for protein. On the other hand, policies to combat climate change require more natural environment conservation, thus demanding less area for animal protein production. In this scenario, increasing the productivity of areas already used for animal protein production is essential to meet the growing demand and to attend to the policies for reducing greenhouse gas emissions, without increasing pasture area. To achieve this goal, the development of more productive cultivars by efficient forage breeding methodologies can help reduce the productivity gap [3].

Tillers are small units of forage grass plants responsible for pasture production. After defoliation of the pasture (e.g., grazing by animals) the regrowth of tillers is crucial to maintain pasture stability and productivity [4,5]. The tillers that effectively contribute to productivity are those that regrow up to eight days after mechanical defoliation or grazing by animals [6]. Thus, one way to measure productivity is to estimate regrowth seven days after defoliation [7]. However, in situ measurements of this trait can be time-consuming, labor-intensive, and is a subjective task. Thus, the development of low-cost technologies for automated plant phenotyping could help scientists and professionals in forage breeding programs. Machine and deep learning combined with mobile devices, such as smartphones, are powerful and low-cost tools for this purpose. The development of such tools could induce less labor and time and more accuracy in the phenotyping process in forage breeding programs, leveraging the efficiency of these programs and contributing to the release of improved cultivars used to reduce the productivity gap.

Many machine learning methods, such as Support Vector Machine (SVM) and K-nearest neighbors (KNN), have been employed and show outstanding results, indicating their potential role in the future of High-Throughput Phenotyping (HTP) [8,9]. Deep Learning is a subset of machine learning techniques known as a versatile tool capable of automatically extracting features and assimilating complex data using a deep neural network. Convolutional Neural Networks (CNNs) have made remarkable achievements in Computer-vision-related tasks [10]. CNN-based approaches have been widely applied to plant phenotyping because of their ability to create robust models that can be embedded in remote sensors [11,12]. The literature often neglects the use of simpler and faster digital image processing approaches. However, in the problem tackled in this study, several research papers have already compared digital image processing and deep learning to grass-like plants, especially between 2018 and 2019, where, in most cases, deep learning showed better performance [13,14,15,16].

Regarding tiller estimation, Zhifeng et al. [17] showed that Magnetic Resonance Imaging (MRI) could be used to measure rice tillers, as well as the conventional X-ray computed tomography system. Yet, an image processing procedure is still necessary. Fang et al. [18] proposed an automatic wheat tiller counting method under field conditions with terrestrial Light Detection and Ranging (LiDAR) using an adaptive layering and hierarchical clustering. Boyle et al. [19] conducted experiments using RGB images of wheat on different days and at three different angles and used a computer vision algorithm based on the Frangi filter. Deng et al. [20] trained a Faster R-CNN on three different backbones (ZFNet, VGGNet16, and VGG-CNN-M-1024) and evaluated productive rice tillers detection using mobile images. They achieved good accuracy compared to manual counting. Kristsis et al. [21] present a plant identification dataset with 125 classes of vascular plants in Greece, which include leaf, flower, fruit, stem in a tree, herb, and fern-like form. They focused the proposal on finding deep learning architectures to deploy on mobile devices. This problem has a different goal from our study. We are not concerned with finding a lightweight architecture. Our proposal aims to help HTP find the best genetic material using mobile images, where computational cost is significant but not a critical factor in our application purposes. In addition, they report their results using validation sets and not as test set [22]. Another interesting result from a grass-like image input can be found in Fujiwara et al. [23]. The authors use a CNN to estimate legume coverage with Unmanned Aerial Vehicle (UAV) imageries. This study samples image patches and estimates the coverage of timothy, white clover, and background using a fine-tuned model for each patch. They evaluate only on GoogLeNet [24].

Although we can find a rich literature in grass-like deep learning literature, to the best of our knowledge, no studies were found that investigate deep-learning-based methods to estimate the regrowth density of tillers in tropical forages using mobile phone images. Mobile phones are more accessible to most researchers than sources used in previous works (e.g., MRI and LiDAR). Furthermore, while other studies count the number of tillers [17,18,19,20], we use a score between 10 and 100 to represent a percentage of regrown tillers to select the top-k best genetic material.

The selection of top-k genotypes requires a scoring function to define the total order. Therefore the natural choice to perform this task is to treat it as a regression problem. If we train the model as a classification problem as classes of 10, 20, 30, all the way to 100, we tie the scores between these ranges, and therefore we lose the fine grain that is very important to select the top-k plants. Treating this problem as a classification problem instead of a regression problem would throw away all the potential of the total ordering possible using scores as the main output of deep learning models. Furthermore, evaluating the use of mobile phones involves two problems: (1) mobile images and (2) small models. The first problem can greatly vary when considering image quality, light, and resolution. The latter considers small models that often compromise accuracy to obtain a lighter model. We compared small models with bigger models to verify the loss acceptable in these applications.

Our objective is to explore deep learning regression-based methods on mobile phone images to assess the regrowth of tillers. Furthermore, different from other studies that directly count the number of tillers, we propose a methodology to assess the percentage of regrown tillers using scores from 10 to 100. We collected 1124 images with two distinct mobile phones and labeled them manually. Six different architectures were evaluated using 10-fold cross-validation with and without transfer learning. We presented a quantitative and qualitative analysis for regression. Thus, our work indicates the potential of the proposed methodology for the tiller regrowth estimation, which will be useful in increasing the efficiency of the breeding program. Our work can be used to build powerful tools for scientists and researchers to evaluate and select the best cultivar candidates in forage breeding programs and contribute to increasing animal protein productivity.

The rest of this paper is organized as follows. Section 2 presents the materials and methods adopted in this study. Section 3 presents the results obtained in the experimental analysis. Section 4 discusses our achievements. Finally, Section 5 summarizes the main conclusions and points to future works.

## 2. Materials and Methods

**Figure 1:**
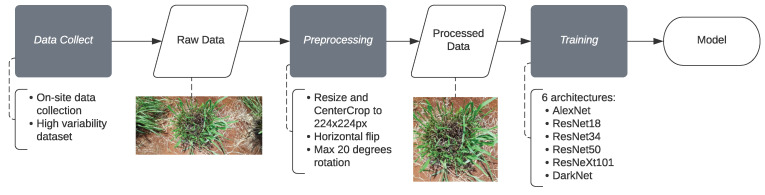
Workflow diagram of the adopted procedure.

We adopt a standard workflow (see Figure 1) of data collection, preprocessing, and training procedures.

### 2.1. Study Area and Dataset

**Figure 2:**
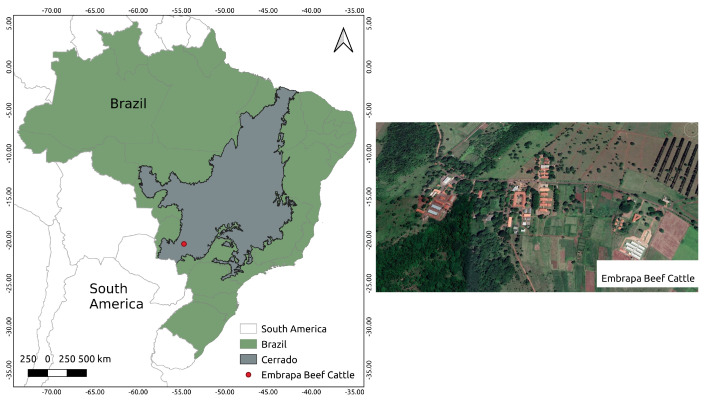
Location of the study area and image of the Embrapa Beef Cattle.

The study was developed in the field at Embrapa Beef Cattle, Campo Grande, Mato Grosso do Sul, Brazil, in the Cerrado Biome (Figure 2). Embrapa Beef Cattle holds the main *Panicum maximum* germplasm bank in the country and is responsible for its breeding program [3]. *Panicum maximum* (Guinea grass) is one of the most important tropical forage grasses because of its high production potential, nutritive value, adaptation ability to different soils and climates, and potential as an alternative source of energy [25,26,27]. Our experiments were conducted in two trials (P7 and P8) of a biparental population of Guinea grass with 210 genotypes showing a high genetic diversity.

The dataset was generated with images obtained with two mobile phones—a Redmi Note 8 Pro and a Moto G4 Play—using the Field Book app [28] that organizes the images and their traits in a CSV file. Our dataset is composed of 1124 labeled images. Table 1 and Table 2 show the number of images collected by date, mobile phone, and experimental area. Each acquisition was close to 1 hour, with a variation of 10 min. The P8 trial was imaged in just one day with a single cell phone, while the P7 trial was imaged on three different days, one day with two cell phones. Considering the different dates (four different days from two seasons—spring and summer) and times (10 a.m. to 11 a.m. and 1 p.m. to 2 p.m.) the images were taken, an attempt was made to generate a dataset with high luminosity variability, making the model more generic and robust. All assessments were made seven days after harvest.

**Figure 3:**
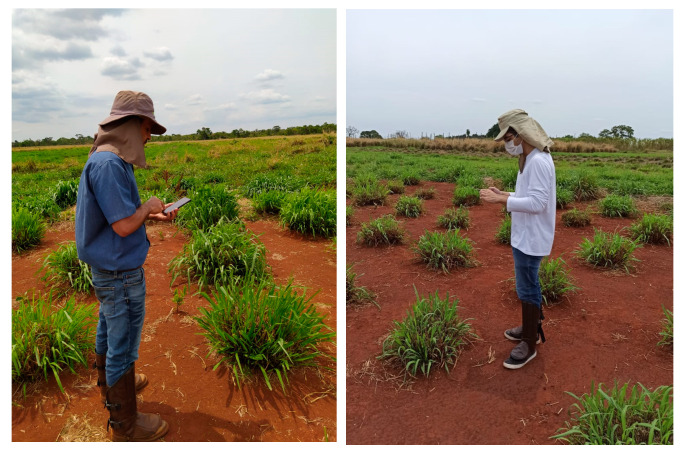
Data collection.

**Figure 4:**
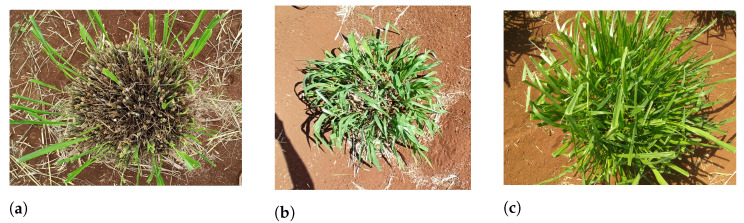
Dataset examples with their respective ground-truth value: (**a**) Regrowth density of 10. The image was taken with Redmi Note 8 Pro. (**b**) Regrowth density of 50. The image was taken with Redmi Note 8 Pro. (**c**) Regrowth density of 100. The image was taken with Moto G Play.

Images taken with Redmi Note 8 Pro are 3264 × 1504 pixels in dimension, and Moto G4 Play’s images are 3264 × 2448 in dimension. They were taken at 1.05m approximately. Figure 3 shows the in situ data collection, while Figure 4 shows samples of different regrowth density from our dataset.

The regrowth density was evaluated in each plot seven days after the mechanical harvest when the regrowth density shows a higher correlation with the next harvest production. To achieve high reliability, the regrowth density measurements must be performed by the same expert (researcher or technical staff) repeatedly after a series of harvests in a year and in different years. For this study, the ground truth data were collected in the field by an Embrapa Beef Cattle researcher (Figure 3). The regrowth was annotated as an integer score dividable by 10, varying from 10 to 100 (included). A score of 10 corresponds to a tiller regrowth of 0% to 10%, and 100 corresponds to a tiller regrowth of 90% to 100%. The literature usually uses a coarser scoring range from 1 to 5, where 1 represents a regrowth of 0% to 20% of tillers, 2 a regrowth from 20% to 40%, 3 a regrowth from 40% to 60%, 4 a regrowth from 60% to 80%, and 5 the regrowth from 80% to 100% [26]. However, we used a more refined scale to have more robustness in our work .

### 2.2. Deep Learning Approach

After the labeled data were organized, we approached the problem using regression with the FastAi library [29]. The Experiment was evaluated with 6 architectures: AlexNet [30], ResNet [31] (18, 34 and 50 layers), ResNeXt101 [32] and DarkNet [33]. We used the AlexNet, ResNet, and ResNeXt implementations from PyTorch [34]. For DarkNet, a repository implementation was used [35]. While using ResNeXt on FastAi, a pre-trained model library was used [36]. In addition, all the architectures were evaluated with a pre-trained model on ImageNet [30] in order to assess the influence of fine tuning.

We performed all experiments using 10-fold cross-validation with an internal hold-out procedure to create training, validation, and test sets. Each fold was divided considering 81% for training, 9% for validation, and 10% for testing. All results presented in this paper were evaluated on the test set. We trained our models on Tesla K80 GPU.

#### 2.2.1. Experimental Setup

**Figure 5:**
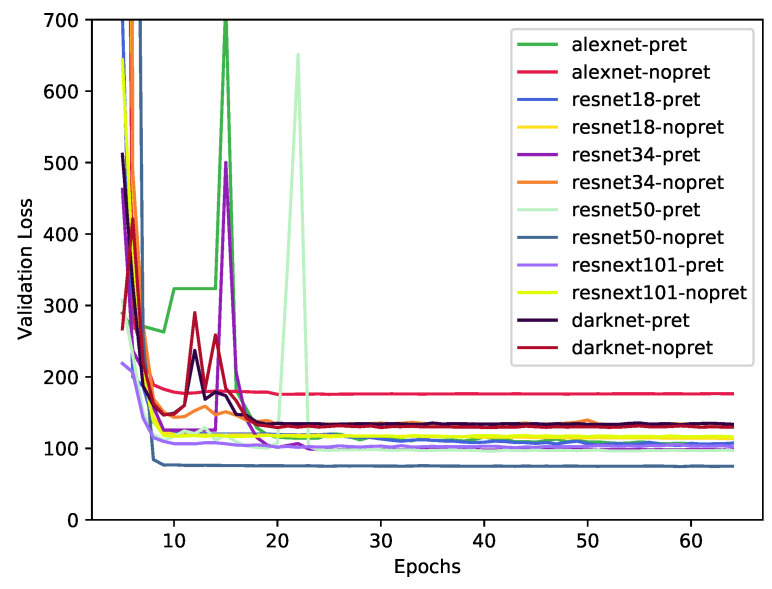
Average of the 10-fold validation loss for each epoch.

We resized the images to 224 × 224 pixels, applying random horizontal flip and max rotation of 20 degrees; both with a probability of 0.75. We trained for 65 epochs (see Figure 5). The training epochs were split into four stages of 10, 10, 5, and 40 epochs. For the first three stages, we used One Cycle Policy [37], and for the last stage, we used the standard training policy. The learning rate was chosen empirically, using the learning rate finder implemented on the FastAi library.

In pre-trained models, after the first stage, we unfroze the third-to-last layer, and after the second stage, we unfroze the whole model. The loss function used was mean square error flat.

At the inference, the predictions were rounded to the closest multiple of 10 between 10 and 100 (included). Table 3 shows how we divided our experiments regarding its architecture, pre-training status, and batch size. The hashtag (#) indicates the experiment number.

#### 2.2.2. Approach Evaluation and Statistical Analysis

We evaluated all of our experiments on Mean Absolute Error (MAE) , Root Mean Square Error (RMSE), Mean Absolute Error (MAPE), and Pearson Correlation (R) and plotted its confusion matrix. Each metric was calculated with the following equations:

(1) $$\mathrm{MAE}=\sum_{i=1}^{n}\left|{\hat{y}}_{i}-y_{i}\right|\frac{1}{n}$$

(2) $$\mathrm{RMSE}=\sqrt{\sum_{i=1}^{n}{\left({\hat{y}}_{i}-y_{i}\right)}^{2}\frac{1}{n}}$$

(3) $$\mathrm{MAPE}=\sum_{i=1}^{n}\left|\frac{y_{i}-{\hat{y}}_{i}}{y_{i}}\right|\frac{100}{n}$$

(4) $$R=\frac{\sum_{i=1}^{n}\left(y_{i}-m_{y}\right)\left({\hat{y}}_{i}-m_{\hat{y}}\right)}{\sum_{i=1}^{n}{\left(y_{i}-m_{y}\right)}^{2}\sum_{i=1}^{n}{\left({\hat{y}}_{i}-m_{\hat{y}}\right)}^{2}}$$

The *y* represents the true value while the $\hat{y}$ represents the predicted value. $m_{y}$ and $m_{\hat{y}}$ are the average of the true values and the average of the predicted values, respectively.

Nonetheless, these metrics do not give better notions of lower and higher values dominance than the ground truth data. So, we were motivated to use Regression Receiver Operating Characteristic (RROC) [38]. RROC space is a plot that depicts the total under-estimation (always negative) against the total over-estimation (always positive). Thus, the closer the point is from (0,0), called RROC heaven, the better the model is. There is a diagonal dashed line *UNDER + OVER* = 0 that represents the points where the under-estimation matches the over-estimation, making the model unbiased. We also used a histogram to evaluate how well the model distribution was learned by comparing it with the true distribution. Finally, we applied the Grad-CAM [39] visual approach.

## 3. Results

Initially, we plotted the loss curve of the models in the validation set in Figure 5. The plots show the validation loss versus the number of epochs. A point is calculated as the average loss over the folds in each epoch. The loss curve gives an overview of the training behavior of the models, and it is possible to check whether an incorrect setting of epochs affected a model result. We can see that all models converged and reached a stable line in the validation set after iteration 30. Another important observation is that no model had potholes in its loss curve, suggesting that early termination might affect the results.

### 3.1. Standard Metrics: MAE, RMSE, MAPE, Pearson Correlation, and Confusion Matrix

Table 4 shows the mean and standard deviation of the mean absolute error, root mean square error, mean absolute percentage error, and Pearson correlation over the 10-fold cross-validation of each attempt. The experiment number refers to Table 3.

Regarding the standard evaluation, the top result, seen in experiment resnet50-pret, has an average MAE of 7.70 and an average RMSE of 10.97; however, the non-pre-trained counterpart did not have such good results. The best couple was ResNeXt101, which achieved an average MAE of 7.72 and 7.81 and an average RMSE of 11.02 and 11.04 with and without fine-tuning, respectively. All experiments showed a correlation higher than 0.81.

**Figure 6:**
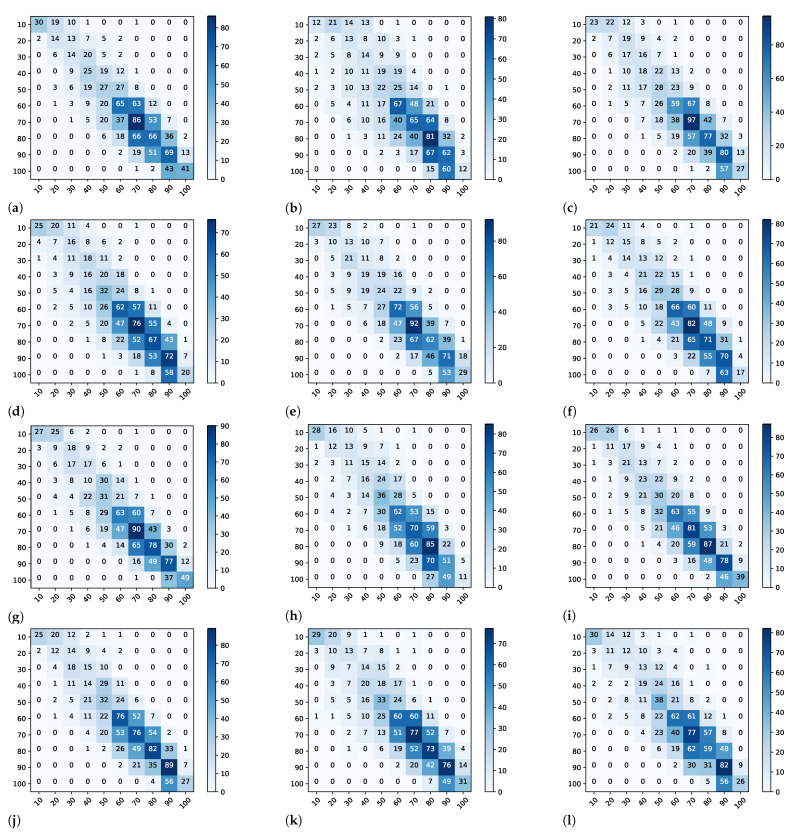
Confusion Matrix plot. The values in the matrices represent the sum of the prediction from all 10 test sets in the 10-fold cross-validation procedure. The lines represent the ground-truth label, and the columns represent the predicted label. (**a**) AlexNet pretrained. (**b**) AlexNet not pretrained. (**c**) ResNet18 pretrained. (**d**) ResNet18 not pretrained. (**e**) ResNet34 pretrained. (**f**) ResNet34 not pretrained. (**g**) ResNet50 pretrained. (**h**) ResNet50 not pretrained. (**i**) ResNeXt101 pretrained. (**j**) ResNeXt101 not pretrained. (**k**) DarkNet pretrained. (**l**) DarkNet not pretrained.

**Figure 7:**
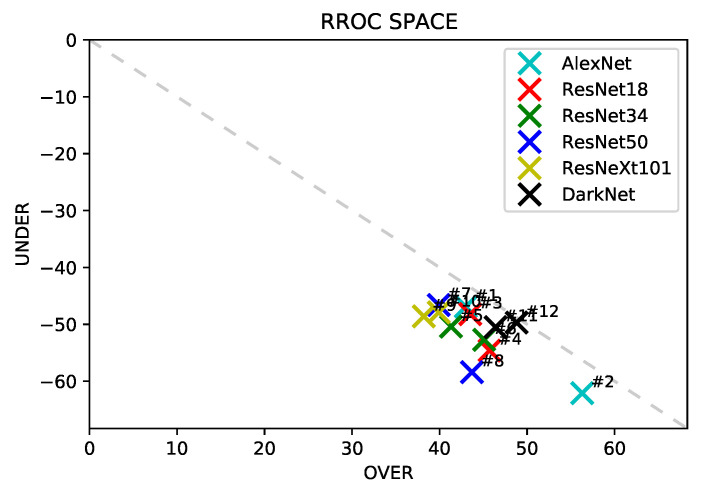
Regression Receiver Operating Characteristic (RROC). The experiments are represented by their number according to Table 3.

**Figure 8:**
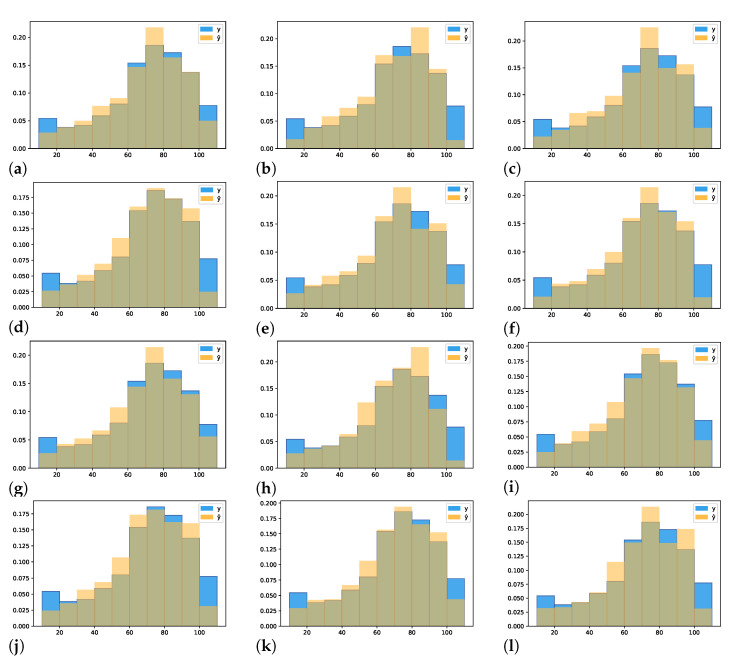
Comparison of prediction $\hat{y}$ vs. real *y* data discrete probability density distribution. (**a**) AlexNet pretrained. (**b**) AlexNet not pretrained. (**c**) ResNet18 pretrained. (**d**) ResNet18 not pretrained. (**e**) ResNet34 pretrained. (**f**) ResNet34 not pretrained. (**g**) ResNet50 pretrained. (**h**) ResNet50 not pretrained. (**i**) ResNeXt101 pretrained. (**j**) ResNeXt101 not pretrained. (**k**) DarkNet pretrained. (**l**) DarkNet not pretrained.

The predictions used to plot Figure 5, Figure 6, Figure 7 and Figure 8 are computed by concatenating all 10 test set results from the cross-validation procedure. In this way, all predictions have no overlapping results, representing the entire dataset as a test set without leaking data from the training to the test set.

Figure 6 shows the confusion matrix where the ground truth (lines) and predicted data (columns) were compared. The matrix main descending diagonal represents the correct predictions. For instance, in Figure 6a, line 70 represents 209 examples (1 + 5 + 20 + 37 + 86 + 53 + 7), where the True value is 70. These 209 examples were predicted as 30 (1), 40 (5), 50 (20), 60 (37), 70 (86), 80 (53), and 90 (7). Therefore, among 209 examples, 86 were predicted correctly as 70, and the remaining examples were around the correct prediction. The area in the matrices lower than 60 represents forages with low regrowth. The goal of the breeding program is to select plants with the best regrowth, i.e., the ones with higher scores for the trait. Therefore, due to the selection applied in past generations, we expect fewer samples with scores less than 60. When we look at the prediction quality in this region of the top two best performing models, resnet50-pret and resnext101-pret (Figure 6g,i, respectively), we can observe that resnext101-pret shows a slightly blueish color pattern closer to the main descending diagonal than resnet50-pret. This pattern indicates that resnext101-pret performs better for lower scores than resnet50-pret. When we look at lower-performing models, such as alexnet-nopret Figure 6b, the results are spread all over scores lower than 60, and the model starts to hit the main diagonal after 60.

The confusion matrix plot shows some values below and above the descending diagonal. However, it is hard to evaluate whether the algorithms had any tendency to predict higher or lower values than the ground truth. One way to assess the tendency to higher or lower values is using RROC [38].

### 3.2. RROC Space

RROC space is a plot that depicts the total under-estimation (always negative) against the total over-estimation (always positive). Thus, the closer the point is from (0,0), called RROC heaven, the better the model is. There is a diagonal dashed line *UNDER + OVER* = 0 that represents the points where the under-estimation matches the over-estimation, making the model unbiased.

Figure 7 shows the RROC plot of the trained models. We can observe that all of them are under the dashed line, which indicates that the models tend to predict lower values than the ground truth values. This result corroborates with the confusion matrix where the values below the descending diagonal, especially 80, 90, and 100, are usually higher than the values above the descending diagonal.

The experiments resnet50-pret and resnext101-pret are closer to the RROC heaven. The least biased model is the one of experiment darknet-nopret. Comparing with Table 4, we observe that experiments resnet50-pret and resnext101-pret show good results; however, the RROC space analysis shows that they are biased. This shows the importance of this analysis, as the standard metrics do not show.

### 3.3. Histogram Analysis

**Figure 9:**
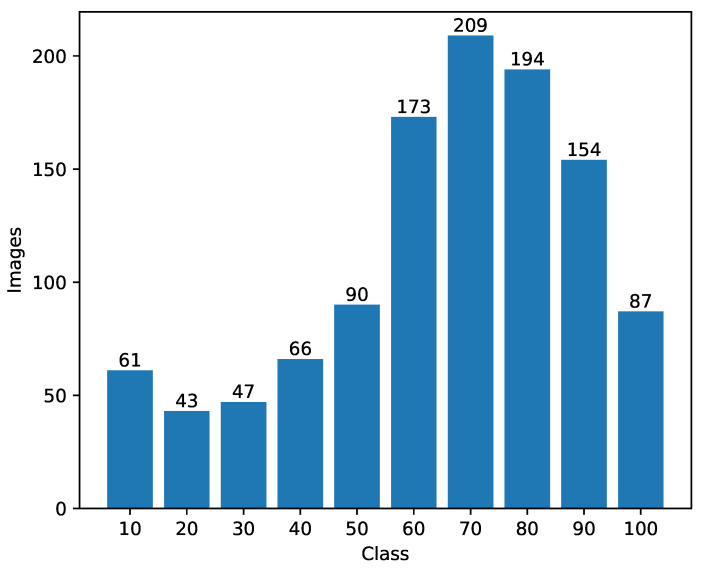
Class attribute *y* distribution.

Figure 8 shows the intersection (greenish color) of the Probability Density Function (PDF) of the ground truth data distribution and the predictions distribution of each experiment. The number of bins is fixed to 10 and represents multiples of 10 between 10 and 100 (included). The *y* distribution is shown in Figure 9.

The intersection area between the distributions in each experiment shown in Table 5 is a numerical representation of the graphs. It allows us to compare the experiments using a numerical score. All the histograms learned to predict the correct distribution well; however, they showed difficulty in predicting the classes well in the end. The best histograms are from the experiment with alexnet-pret, resnext101-pret, and darknet-pret which achieved 0.93 of intersection area between both distributions. We used the Kullback–Leibler divergence (KL divergence) to measure the distance between both probability distributions. The distributions most similar to ground truth data are from experiments with alexnet-pret, resnext50-pret, and darknet-pret.

### 3.4. Visual Inspection

**Figure 10:**
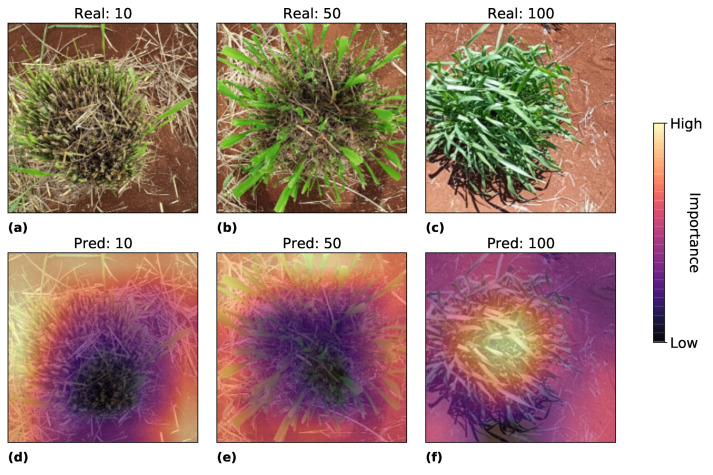
Grad-Cam analysis of the best predictions. Figures (**a**–**c**) represents the real image with true score of 10, 50 and 100. Figures (**d**–**f**) represent the activation maps where the model predicted 10, 50, 100.

**Figure 11:**
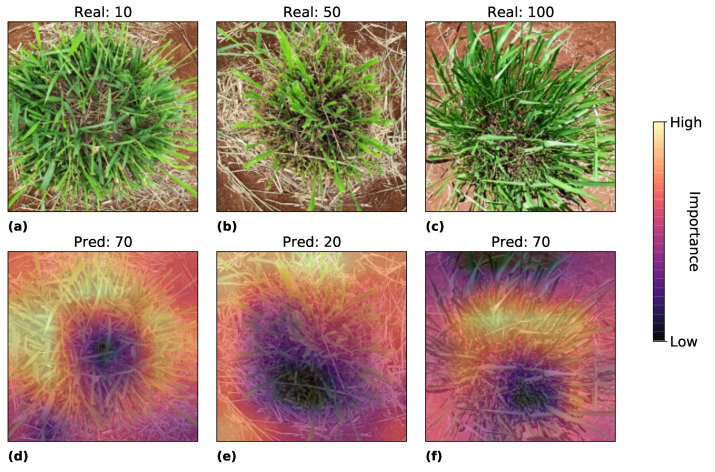
Grad-Cam analysis of the worst predictions. Figures (**a**–**c**) represents the real image with true score of 10, 50 and 100. Figures (**d**–**f**) represent the activation maps where the model predicted 70, 20, 70.

Experiment resnet50-pret shows the top MAE, RMSE, and correlation among the algorithms tested. We analyzed the image regions that this model considers more discriminating to define the regrowth areas, i.e., where the model looks at the image to predict the regrowing areas. For this, we look at the last activation map in the model using Grad-CAM. Figure 10 and Figure 11 show the heatmap of Grad-CAM on Experiment resnet50-pret for the best and worst prediction for 10, 50, and 100 ground truth values, respectively. Warmer colors indicate areas that played the most important role in the model’s decision, while colder colors mean the opposite.

The heatmap in Figure 10 shows a pattern where, in the lower density (regrowth score 10), the model avoids looking at the center of the plot and focuses on the border of the plot, leaving a circle in the middle where the model does not analyze. With the high density (regrowth score 100), the heatmap is stronger in an opposite way, focusing more on the center of the plot. This result corroborates with common sense that the high-density plot has more leaves in the center where the model looks.

When looking at Figure 11a, the image seems to be mislabeled to 10. We can see from the image that the plot presented a relatively acceptable regrowth and much better than Figure 10a, and we believe that the model predicted a better score than the ground truth. The same occurs in the other images where the prediction seems better than the ground truth. The pattern of higher regrowth is similar to Figure 10, where the higher the regrowth, the more critical the center of the plot is.

### 3.5. Efficiency Analysis

We analyzed the efficiency of the experiments by comparing the average time a model takes to compute a single example. Table 6 shows the number of parameters for each experiment and the average inference time on GPU (tested on Tesla M4) and CPU. We picked 112 examples of our dataset for this analysis. As expected, the models are much faster on GPU than on CPU. Therefore, GPU is preferable to CPU. However, in our case, the time spent by inference is not an issue because we do not need the prediction in real-time, and even the slowest model (resnext101-pret) is already quite fast.

## 4. Discussion

This study estimates the regrowth density of tropical forages using mobile phone images. To achieve such a goal, we evaluated a series of standard and state-of-the-art deep learning methods from a simpler model such as AlexNet with only five layers to a more complex model such as ResNext101 with 101 layers. These models were adapted to tackle the problem as a regression problem.

For the first time, we report that deep learning methods can deliver correlations from 0.81 to 0.89 in estimating the regrowth density using mobile phone images. We believe that this result is very acceptable and has the potential to speed up data collection of regrowth density and consequently increase the efficiency of forage breeding programs. The closest approach found in the literature was the study conducted by Deng et al. [20] for rice tillers. The authors used a completely different approach. Their approach required harvesting the rice and evaluating the cross-sections of rice tillers. Using object detection, they estimated the number of productive tillers. Our approach requires just a plot image obtained from a mobile phone without harvesting or other labor-intensive intervention.

Deeper neural nets perform better than the shallower version of the same architecture in most problems [31]. In HTP, we found some controversy where the deeper model di not always produce the best result. The study conducted by Oliveira et al. [40] using aerial images taken by an Unmanned Aerial Vehicle (UAV) showed some results where the best performing model among AlexNet, ResNeXt50, MaCNN, LF-CNN, and DarkNet53 was a simple AlexNet. Intrigued by these results, we evaluated a broader range of deep learning architectures with a more diverse number of layers. Interestingly, a 50-layer (Resnet50) network achieved our best-performing result. Again, in a traditional computer vision task, we expected the 101 layer network to give the best result, which did not occur.

The analysis using RROC indicated that all models were below the descending diagonal, suggesting that deep learning models tend to undervalue the prediction of the results in the problem setting of this paper. Castro et al. [41] also plotted RROC in a biomass prediction problem using deep learning and aerial images, and in their results, this tendency did not exist. We believe that this tendency happens due to the skewed data distribution (Figure 9) toward higher values.

The heatmap results shed light on where the network is “looking” to predict the regrowth density. To the best of our knowledge, this result is the first study to address the interpretability of deep learning models on regrowth. The results indicate that the circular region is the main area to reveal the lower regrowth area. The center of the plot is the most characteristic area for higher regrowth images in deep learning.

Compared to similar works, ours differs for not using any complex sensor technology, such as MRI and LiDAR, which are highly priced and excessive compared to a mobile phone. In addition, there is no need for a scheme to take pictures on different days and rotations and handcraft features. Furthermore, the main distinction from other works is the estimated trait. We calculated a score representing the regrowth percentage of the tillers instead of counting the number of tillers.

The use of machine learning must be used with care. Although the proposed approach can give valuable estimates of tiller regrowing, it is not advisable to completely substitute the manual labeling field regrow density. It is always good to collect smaller validation sets to evaluate if the learned models still give good estimates. Therefore, the proposed approach never intended to completely replace the manual labeling of fields but rather to allow the HTP research to multiply the number of plots while reducing the need for manual labeling collection.

## 5. Conclusions

To the best of our knowledge, this is the first research that evaluated CNN-based architectures to estimate regrowth density using RGB images collected by mobile phones. From our perspective, this study also presents the following contributions according to our results: (1) deep learning can deliver correlations from 0.81 to 0.89 in estimating the regrowth density using mobile phone images; (2) the best-performing architecture is not always the deeper model for this problem; (3) the deep learning models tend to undervalue the predictions in our problem setting and; (4) the heatmap indicates the patterns that deep learning models use to predict regrowth density.

Previous works focus on estimating the tiller number. We used a score that represents the percentage of regrown tillers, and we collected a dataset with images of forages taken on different days, locations, phones, and genotypes, promoting more generalized models.

Our results indicate that we might succeed in using our methods for new data prediction. To develop new cultivars, the researchers need to evaluate and select for multiple traits in the breeding program. Thus, there is a huge consumption in time and cost, sometimes with low accuracy, for performing the phenotyping step. Thus, training new algorithms to estimate traits such as disease and insect damages, mineral deficiencies, seed number, and other traits is the next step of this work for using deep learning associated with low-cost mobile devices.

In future work, we will evaluate the problem by employing lightweight deep learning architectures to deploy the model inside the mobile phone. In this way, the annotators can speed up their labeling process, and their task is more related to validating the predictions and collecting images than labeling the plot. We also plan to evaluate the problem using the Learning-To-Rank algorithm and evaluate the use of UAV-based images.

## Figures and Tables

**Table 1:** Images collected with each mobile phone at P7.

Date	Moto G4 Play	Redmi Note 8 Pro	Time
30 October 2019	225	225	10 a.m.
10 December 2019	0	225	10 a.m.
18 February 2020	0	225	10 a.m.

**Table 2:** Images collected with each mobile phone at P8.

Date	Moto G4 Play	Redmi Note 8 Pro	Time
18 February 2020	0	224	1 p.m.

**Table 3:** Experimental setup.

#	Experiment	Model	Pretrained	Batch Size	Epochs
1	alexnet-pret	AlexNet	Yes	128	65
2	alexnet-nopret	AlexNet	No	128	65
3	resnet18-pret	ResNet18	Yes	128	65
4	resnet18-nopret	ResNet18	No	128	65
5	resnet34-pret	ResNet34	Yes	128	65
6	resnet34-nopret	ResNet34	No	128	65
7	resnet50-pret	ResNet50	Yes	64	65
8	resnet50-nopret	ResNet50	No	64	65
9	resnext101-pret	ResNeXt101	Yes	32	65
10	resnext101-nopret	ResNeXt101	No	32	65
11	darknet-pret	DarkNet	Yes	32	65
12	darknet-nopret	DarkNet	No	32	65

**Table 4:** Mean and standard deviation for each model setup of the 10-fold cross-validation. Best results presented in bold font.

Experiment	MAE	RMSE	MAPE	Correlation (*r*)
alexnet-pret	8.00 ± 1.84	11.26 ± 2.10	17.94 ± 4.61	0.88 ± 0.03
alexnet-nopret	10.53 ± 2.20	14.30 ± 2.71	26.51 ± 3.74	0.81 ± 0.04
resnet18-pret	8.16 ± 1.86	11.43 ± 1.95	19.37 ± 4.02	0.88 ± 0.03
resnet18-nopret	8.92 ± 2.35	12.24 ± 2.65	20.71 ± 5.24	0.86 ± 0.04
resnet34-pret	8.16 ± 1.92	11.35 ± 2.22	18.61 ± 5.01	0.88 ± 0.04
resnet34-nopret	8.70 ± 2.14	12.00 ± 2.44	20.44 ± 4.92	0.87 ± 0.04
resnet50-pret	**7.70 ± 1.88**	**10.97 ± 2.28**	17.83 ± 4.44	**0.89 ± 0.04**
resnet50-nopret	9.08 ± 2.26	12.37 ± 2.70	20.71 ± 5.80	0.86 ± 0.04
resnext101-pret	7.72 ± 2.44	11.02 ± 2.56	**17.70 ± 5.82**	**0.89 ± 0.04**
resnext101-nopret	7.81 ± 2.00	11.04 ± 2.16	18.55 ± 5.19	**0.89 ± 0.04**
darknet-pret	8.62 ± 2.17	12.13 ± 2.60	19.80 ± 4.89	0.86 ± 0.04
darknet-nopret	8.76 ± 2.08	12.21 ± 2.26	20.40 ± 6.07	0.86 ± 0.05

**Table 5:** Intersection areas of the histogram shown in Figure 8. Best results presented in bold.

Experiment	KL Divergence	Intersection Area
alexnet-pret	**0.02**	**0.93**
alexnet-nopret	0.10	0.88
resnet18-pret	0.05	0.89
resnet18-nopret	0.05	0.92
resnet34-pret	0.03	0.91
resnet34-nopret	0.07	0.91
resnet50-pret	**0.02**	0.92
resnet50-nopret	0.10	0.88
resnext101-pret	0.03	**0.93**
resnext101-nopret	0.05	0.91
darknet-pret	**0.02**	**0.93**
darknet-nopret	0.04	0.90

**Table 6:** Efficiency Analysis.

Experiment	Number of Params	Inference Time (GPU) (ms)	Inference Time (CPU) (ms)
ine alexnet-pret	265,217	3.98	34.98
alexnet-nopret	265,217	3.96	34.96
resnet18-pret	537,985	6.86	100.03
resnet18-nopret	537,985	7.06	103.71
resnet34-pret	545,409	10.17	177.52
resnet34-nopret	545,409	10.21	171.52
resnet50-pret	2,160,513	13.21	241.48
resnet50-nopret	2,160,513	13.30	239.66
resnext101-pret	2,245,249	27.26	589.90
resnext101-nopret	2,245,249	27.03	500.84
darknet-pret	40,585,953	15.37	403.45
darknet-nopret	40,585,953	15.33	364.80

## Data Availability

The data that support the findings of this study are available from the corresponding author with the permission of Embrapa.

## Abbreviations

The following abbreviations are used in this manuscript:

MDPI	Multidisciplinary Digital Publishing Institute
CNN	Convolutional Neural Network
DL	Deep Learning
MRI	Magnetic Resonance Imaging

## References

[1] Júnior L., Santos C., Mesquita V., Parente L. Dynamics of Brazilian Pastures: Occupation of Areas and Signs of Degradation-2010 to 2018. https://www.gov.br/agricultura/pt-br/assuntos/noticias/estudo-mostra-reducao-de-26-8-milhoes-de-hectares-de-pastagens-degradadas-em-areas-que-adotaram-o-plano-abc/Relatorio_Mapa1.pdf.

[2] Strassburg B., Latawiec A., Barioni L., Nobre C., Silva V., Valentim J., Vianna M., Assad E. (2014). When enough should be enough: Improving the use of current agricultural lands could meet production demands and spare natural habitats in Brazil. Glob. Environ. Chang..

[3] Jank L., Barrios S., Valle C., Simeão R., Alves G. (2014). The value of improved pastures to Brazilian beef production. Crop. Pasture Sci..

[4] Silva C., Gimenes F., Gomes M., Berndt A., Gerdes L. (2012). Tiller population density and tillering dynamics in marandu palisade grass subjected to strategies of rotational stocking management and nitrogen fertilization. Pasture Forage Util..

[5] Garay A.H., Hodgson M. (1999). Tiller size/density compensation in perennial ryegrass miniature swards subject to differing defoliation heights and a proposed productivity index. Grass Forage Sci..

[6] Corsi M. (1984). Effects of Nitrogen Rates and Harvesting Intervals on Dry Matter Production, Tillering and Quality of the Tropical Grass, Panicum Maximum, Jacq. https://repositorio.usp.br/item/000742995.

[7] Jank L. (1995). Melhoramento e seleção de variedades de Panicum maximum. SimpóRio Sobre Manejo Pastagem.

[8] Singh A., Ganapathysubramanian B., Singh A.K., Sarkar S. (2016). Machine Learning for High-Throughput Stress Phenotyping in Plants. Trends Plant Sci..

[9] Mochida K., Koda S., Inoue K., Hirayama T., Tanaka S., Nishii R., Melgani F. (2018). Computer vision-based phenotyping for improvement of plant productivity: A machine learning perspective. GigaScience.

[10] Al-Saffar A.A.M., Tao H., Talab M.A. Review of deep convolution neural network in image classification. Proceedings of the 2017 International Conference on Radar, Antenna, Microwave, Electronics, and Telecommunications (ICRAMET).

[11] Singh A.K., Ganapathysubramanian B., Sarkar S., Singh A. (2018). Deep Learning for Plant Stress Phenotyping: Trends and Future Perspectives. Trends Plant Sci..

[12] Jiang Y., Li C. (2020). Convolutional Neural Networks for Image-Based High-Throughput Plant Phenotyping: A Review. Plant Phenomics.

[13] Kounalakis T., Triantafyllidis G.A., Nalpantidis L. (2019). Deep learning-based visual recognition of rumex for robotic precision farming. Comput. Electron. Agric..

[14] Zhang X., Han L., Dong Y., Shi Y., Huang W., Han L., González-Moreno P., Ma H., Ye H., Sobeih T. (2019). A deep learning-based approach for automated yellow rust disease detection from high-resolution hyperspectral UAV images. Remote Sens..

[15] Liu T., Abd-Elrahman A., Morton J., Wilhelm V.L. (2018). Comparing fully convolutional networks, random forest, support vector machine, and patch-based deep convolutional neural networks for object-based wetland mapping using images from small unmanned aircraft system. Gisci. Remote Sens..

[16] Yoo C., Han D., Im J., Bechtel B. (2019). Comparison between convolutional neural networks and random forest for local climate zone classification in mega urban areas using Landsat images. Isprs. J. Photogramm. Remote Sens..

[17] Zhi-feng H., Liang G., Cheng-liang L., Yi-xiang H., Qing-liang N. (2016). Measurement of Rice Tillers Based on Magnetic Resonance Imaging. Ifac. Papersonline.

[18] Fang Y., Qiu X., Guo T., Wang Y., Cheng T., Zhu Y., Chen Q., Cao W., Yao X., Niu Q. (2020). An automatic method for counting wheat tiller number in the field with terrestrial LiDAR. Plant Methods.

[19] Boyle R., Corke F., Doonan J. (2015). Automated estimation of tiller number in wheat by ribbon detection. Mach. Vis. Appl..

[20] Deng R., Jiang Y., Tao M., Huang X., Bangura K., Liu C., Lin J., Qi L. (2020). Deep learning-based automatic detection of productive tillers in rice. Comput. Electron. Agric..

[21] Kritsis K., Kiourt C., Stamouli S., Sevetlidis V., Solomou A., Karetsos G., Katsouros V., Pavlidis G. (2021). GRASP-125: A Dataset for Greek Vascular Plant Recognition in Natural Environment. Sustainability.

[22] Cawley G.C., Talbot N.L. (2010). On over-fitting in model selection and subsequent selection bias in performance evaluation. J. Mach. Learn. Res..

[23] Fujiwara R., Nashida H., Fukushima M., Suzuki N., Sato H., Sanada Y., Akiyama Y. (2022). Convolutional neural network models help effectively estimate legume coverage in grass-legume mixed swards. Front. Plant Sci..

[24] Szegedy C., Liu W., Jia Y., Sermanet P., Reed S., Anguelov D., Erhan D., Vanhoucke V., Rabinovich A. Going deeper with convolutions. Proceedings of the IEEE Conference on Computer Vision and Pattern Recognition.

[25] de Lima Veras E.L., Difante G.d.S., Chaves Gurgel A.L., Graciano da Costa A.B., Gomes Rodrigues J., Marques Costa C., Emerenciano Neto J.V., Gusmão Pereira M.D., Ramon Costa P. (2020). Tillering and Structural Characteristics of Panicum Cultivars in the Brazilian Semiarid Region. Sustainability.

[26] Braz T., Martuscello J., Jank L., Fonseca D., Resende M., Evaristo A. (2017). Genotypic value in hybrid progenies of Panicum maximum Jacq. Ciência Rural.

[27] Jank L., Lima E., Simeão R., Andrade R. (2013). Potential of Panicum maximum as a source of energy. Trop. Grasslands-Forrajes Trop..

[28] PhenoApps Field Book. https://github.com/PhenoApps/Field-Book.

[29] Howard J., Gugger S. (2020). Fastai: A Layered API for Deep Learning. Information.

[30] Krizhevsky A., Sutskever I., Hinton G.E. (2012). ImageNet classification with deep convolutional neural networks. Commun. ACM.

[31] He K., Zhang X., Ren S., Sun J. Deep Residual Learning for Image Recognition. Proceedings of the IEEE Conference on Computer vision and Pattern Recognition.

[32] Xie S., Girshick R.B., Dollár P., Tu Z., He K. Aggregated Residual Transformations for Deep Neural Networks. Proceedings of the 2017 IEEE Conference on Computer Vision and Pattern Recognition (CVPR).

[33] Redmon J., Divvala S., Girshick R.B., Farhadi A. You Only Look Once: Unified, Real-Time Object Detection. Proceedings of the 2016 IEEE Conference on Computer Vision and Pattern Recognition (CVPR).

[34] Paszke A., Gross S., Massa F., Lerer A., Bradbury J., Chanan G., Killeen T., Lin Z., Gimelshein N., Antiga L., Wallach H., Larochelle H., Beygelzimer A., dAlché Buc F., Fox E., Garnett R. (2019). PyTorch: An Imperative Style, High-Performance Deep Learning Library. Advances in Neural Information Processing Systems 32.

[35] Kwon Y. Darknet53. https://github.com/developer0hye/PyTorch-Darknet53.

[36] Cadene R. Pretrained models for Pytorch. https://github.com/Cadene/pretrained-models.pytorch.

[37] Smith L.N. (2018). A disciplined approach to neural network hyper-parameters: Part 1—Learning rate, batch size, momentum, and weight decay. arXiv.

[38] Hernández-Orallo J. (2013). ROC curves for regression. Pattern Recognit..

[39] Selvaraju R.R., Cogswell M., Das A., Vedantam R., Parikh D., Batra D. Grad-CAM: Visual Explanations from Deep Networks via Gradient-Based Localization. Proceedings of the 2017 IEEE International Conference on Computer Vision (ICCV).

[40] de Oliveira G.S., Marcato Junior J., Polidoro C., Osco L.P., Siqueira H., Rodrigues L., Jank L., Barrios S., Valle C., Simeão R. (2021). Convolutional Neural Networks to Estimate Dry Matter Yield in a Guineagrass Breeding Program Using UAV Remote Sensing. Sensors.

[41] Castro W., Marcato Junior J., Polidoro C., Osco L.P., Gonçalves W., Rodrigues L., Santos M., Jank L., Barrios S., Valle C. (2020). Deep learning applied to phenotyping of biomass in forages with UAV-based RGB imagery. Sensors.

